# Altered Pharmacokinetics Parameters of Vancomycin in Patients with Hematological Malignancy with Febrile Neutropenia, a Bayesian Software Estimation

**DOI:** 10.3390/antibiotics12060979

**Published:** 2023-05-29

**Authors:** Abdullah M. Alzahrani, Anjum Naeem, Aeshah AlAzmi, Alqassem Y. Hakami, Shahid Karim, Ahmed S. Ali, Fatemah Omer Kamel, Rami M. Alzhrani, Teaf S. Alkhaldi, Loujayne A. Maghrabi, Norah F. Alshehri, Yahya A. Alzahrani

**Affiliations:** 1 Pharmaceutical Care Department, Ministry of National Guard—Health Affairs, Jeddah 22384, Saudi Arabia; ansarian@mngha.med.sa (A.N.); alazmiai@mngha.med.sa (A.A.); 2 King Abdullah International Medical Research Center, Jeddah 21423, Saudi Arabia; hakamial@mngha.med.sa; 3 College of Medicine, King Saud bin Abdulaziz University for Health Sciences, Jeddah 22384, Saudi Arabia; 4 Department of Pharmacology, Faculty of Medicine, King Abdulaziz University, Jeddah 21589, Saudi Arabia; skaled@kau.edu.sa (S.K.); asali@kau.edu.sa (A.S.A.); foakamel@kau.edu.sa (F.O.K.); 5 Department of Pharmaceutics and Industrial Pharmacy, College of Pharmacy, Taif University, Taif 21944, Saudi Arabia; r.zhrani@tu.edu.sa; 6 College of Pharmacy, Taif University, Taif 21944, Saudi Arabia; itzzteva@outlook.com; 7 College of Pharmacy, Umm Al-Qura University, Makkah 21955, Saudi Arabia; 8 Department of Pharmacy, East Jeddah Hospital, Ministry of Health, Jeddah 22253, Saudi Arabia

**Keywords:** augmented renal clearance and vancomycin, febrile neutropenia and augmented renal clearance, vancomycin low trough in hematological malignancy, vancomycin monitoring in the neutropenic patients

## Abstract

The pharmacokinetics of vancomycin vary significantly between specific groups of patients, such as critically ill patients and patients with hematological malignancy (HM) with febrile neutropenia (FN). Recent evidence suggests that the use of the usual standard dose of antibiotics in patients with FN may not offer adequate exposure due to pharmacokinetic variability (PK). Therefore, the purpose of this study is to assess the effect of FN on AUC_0–24_ as a key parameter for vancomycin monitoring, as well as to determine which vancomycin PK parameters are affected by the presence of FN using Bayesian software PrecisePK in HM with FN. This study was carried out in King Abdulaziz Medical City. All adult patients who were admitted to the Princess Norah Oncology Center PNOC between 1 January and 2017 and 31 December 2020, hospitalized and received vancomycin with a steady-state trough concentration measured before the fourth dose, were included. During the trial period, 297 patients received vancomycin during their stay at the oncology center, 217 of them meeting the inclusion criteria. Pharmacokinetic parameters were estimated for the neutropenic and non-FN patients using the precise PK Bayesian platform. The result showed that there was a significant difference (*p* < 0.05) in vancomycin clearance Cl_van_, the volume of distribution at a steady-state V_dss_, the volume of distribution for peripheral compartment V_dp_, half-life for the elimination phase t½_β_, and the first-order rate constant for the elimination process β in FN compared to non-FN patients. Furthermore, AUC_0–24_ was lower for FN patients compared to non-FN patients, *p* < 0.05. FN has a significant effect on the PK parameters of vancomycin and AUC_0–24_, which may require specific consideration during the treatment initiation.

## 1. Introduction

Despite the long history of vancomycin use, many concerns remain about which dosing strategy is best to maximize bactericidal action and avoid toxicity [1]. The PK/PD parameter for therapeutic drug monitoring (TDM) that best describes the safety and efficacy of vancomycin is the ratio of the area under the curve over a 24 h period to the minimum inhibitory concentration (AUC_0–24_/MIC) [2,3]. Considering a MIC ≤ 1 mg/L, recommendations suggest a target AUC_0–24_ of 400–600 mg·h/L [4]. Seeking simplicity and practicality, the 2009 clinical recommendations propose maintaining a minimum vancomycin trough concentration > 10 mg/L and traditionally this has been utilized as an alternative target in clinical practice for AUC_0–24_ ≥ 400 mg·h/L [5]. This method has been argued recently, and several retrospective studies have revealed that trough concentration is not a suitable indicator for AUC_0–24_. Among the disadvantages of the trough-based method is that the dose to obtain a recommended trough concentration results in extreme exposure and an increased probability of nephrotoxicity without improving efficacy [6,7].

Current data support a goal for a daily AUC_0–24_/MIC ratio of 400 mg·h/L [1,4]. A method to calculate AUC_0–24_ required two concentration measurements during the one interval, both of which are acquired to be withdrawn at steady state. Then, using first-order pharmacokinetic equations, AUC_0–24_ can be determined [8]. The difficulty of this strategy is the degree of complexity associated with data entry and computation [4,9]. An alternative method is using Bayesian prediction, which uses specialized software programs that combine observed concentrations with “prior” knowledge collected from population pharmacokinetic models to predict individual pharmacokinetic parameters and drug exposure [4,10]. It has many advantages over other methods of calculation, as it gives high accuracy and can be estimated using only one sample. However, software systems that provide integrated Bayesian modeling are considered costly for hospital deployment, hindering wider clinical practice adoption [11,12].

The pharmacokinetics of vancomycin vary significantly between specific groups of patients, such as critically ill patients [13] and patients with hematological malignancy with febrile neutropenia (FN) [14,15]. Recent evidence suggests that the use of the usual standard dose of antibiotics in patients with FN may not offer adequate exposure due to pharmacokinetic variability (PK) [16]. Furthermore, several studies involving meropenem, ceftazidime piperacillin, and vancomycin have reported that the pharmacokinetic profile in FN patients differs from that in normal healthy patients [17]. The suboptimal concentration of vancomycin trough in neutropenic patients has been extensively discussed elsewhere [15,18].

The existing studies have noted differences in vancomycin disposition in neutropenia patients compared to non-FN patients with or without HM. In neutropenic patients, several studies showed that vancomycin CL tends to be higher than in non-FN patients, with a range of 25–30% [15,19]. On the other hand, there is contradictory evidence as to whether FN will affect V_d_ [20]; such pharmacokinetic alterations during neutropenia require a better understanding of vancomycin pharmacokinetics in those patients to guide dosing and improve clinical outcomes.

While most of the previous studies have been carried out to evaluate the effect of FN on the vancomycin trough concentration, there have been few attempts to investigate this effect on AUC_0–24_. Furthermore, no study has been made to assess which pharmacokinetic parameter is affected by FN [21]. Identifying and quantifying the effect of FN on vancomycin PK parameters and AUC_0–24_ will allow the establishment of a safe and effective dosing regimen for this patient population.

Therefore, the purpose of this study is to assess the effect of FN on AUC_0–24_ as a key parameter for vancomycin monitoring, as well as to determine which vancomycin PK parameters are affected by the presence of FN using Bayesian software PrecisePK in HM with FN. In addition, the current work will examine the clinical discordance between the traditional trough method and the AUC_0–24_ method. In addition, factors that contribute to the estimated AUC_0–24_ will also be identified.

## 2. Results

**Figure 1:**
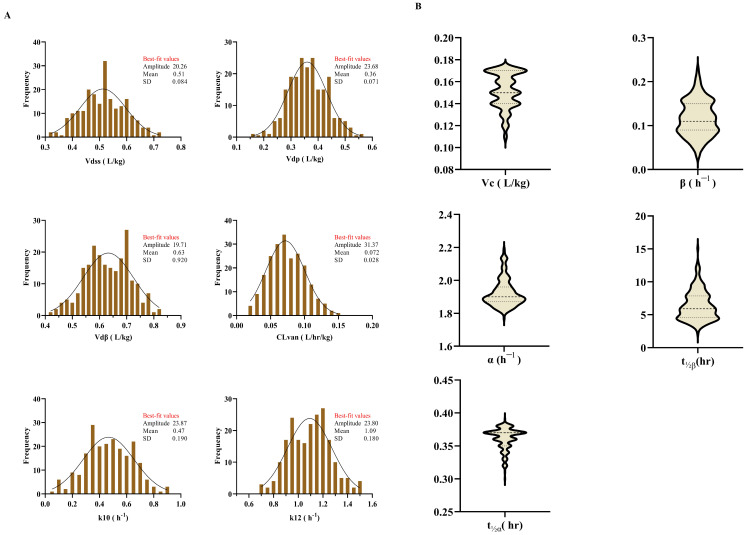
PK data exploratory plots. (**A**) The vancomycin PK profiles for normally distributed data are shown by a histogram while (**B**) the vancomycin PK profiles for remaining data are shown by a violin plot; the center line of the violin represents the group median, while the upper and lower lines inside the violin represent the 75th and 25th percentiles.

During the trial period, 297 patients received vancomycin, 217 meeting the inclusion criteria. Patients in this study were divided into a FN group (n = 158) and a non-FN group (n = 59). The demographic data of the patients are shown in Table 1. Most of the patients were diagnosed with leukemia (64.50%), 28.6% had lymphoma, and the smallest proportion were myeloma patients (6.9%). The mean baseline creatinine clearance (CrCl) in the FN group was 125.99 mL/min, calculated according to the Cockcroft–Gault equation. Augmented renal clearance (ARC) was reported in half of the FN patients and one-quarter of the non-FN patients. The mean vancomycin dose of 31.11 mg/kg/day in FN patients and 30.48 mg/kg/day in the non-FN group. However, the estimated AUC_0–24_ for patients with FN patients was 403 mg·h/L, and 461 mg·h/L for patients without FN. The desired AUC_0–24_ and trough were reported in 84 (38.7%) and 77 (35.5%), respectively, of the total included patient. All exploratory data plots of vancomycin PK profiles are presented in Figure 1.

### 2.1. Vancomycin PK Parameters in the FN and Non-FN Groups

The independent *t*-test demonstrated a significant decrease (*p* < 0.05) in the mean of volume distribution of vancomycin at steady state (V_dss_), the volume of the peripheral compartment (V_p_), and the transfer rate constant from the central to the peripheral compartment (K_12_) in the FN group compared with the non-FN group, with mean values of 0.502 L/kg, 0.352 L/kg, and 1.071 h^−1^ compared to 0.531 L/kg, 0.382 L/kg and 1.15 h^−1^, respectively, while there was a significant increase (*p* < 0.05) in vancomycin clearance (Cl_van_) and the elimination rate constant from the central compartment (K_10_) in the FN patients compared with non-FN patients, with means of 0.078 L/h/kg and 0.504 h^−1^, respectively. Furthermore, a non-significant change (*p* > 0.05) was seen in the volume of vancomycin distribution during the elimination phase (Vd_β_) between the two groups (Table 2).

The Mann–Whitney test illustrated that the half-life for the elimination phase (t½_β_) was statistically significantly lower in the FN (Med = 5.6) than in the non-FN group (Med = 7.4), (U = 2854, *p* < 0.05). FN patients had a statistically significantly higher hybrid first-order rate constant for the elimination process (β) (Med = 0.12) compared to non-FN patients (Med = 0.1); (U = 2805, *p* < 0.05). Additionally, the result showed no significant differences (*p* > 0.05) found between the two groups in terms of half-life for the elimination phase (t½_α_), the hybrid first-order rate constant for the distribution process (α), and the volume of the central compartment (V_c_).

### 2.2. Estimated AUC in the FN and Non-FN Groups and the Percentage of Patients Who Achieved Optimal AUC_0–24_

**Figure 2:**
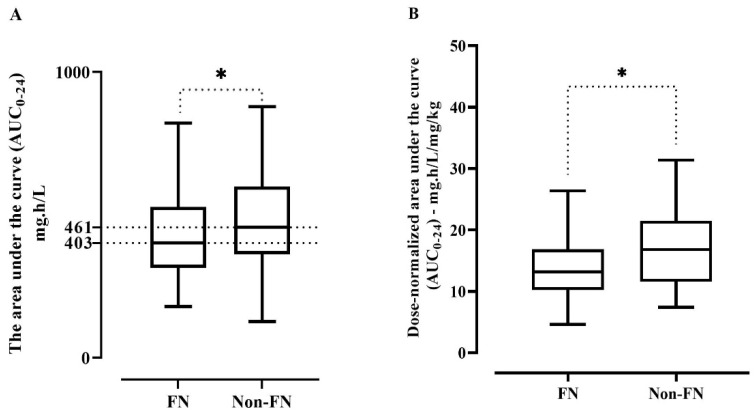
The difference among neutropenic and non-FN patients in (**A**) vancomycin AUC_0–24_; * *p* < 0.05, compared with non-FN patient values; by the Mann–Whitney test. Middle line: median; upper/lower box: upper or lower 25% of data, and (**B**) vancomycin dose-normalized AUC_0–24_; *p* < 0.05 compared with non-FN patient values, via the Student *t*-test. Data are expressed as the mean ± SEM; n = 217.

**Figure 3:**
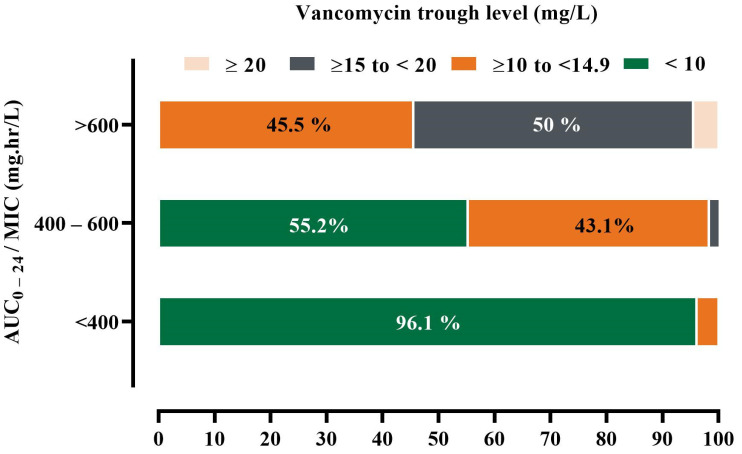
Percentage of FN patients who achieved optimal AUC_0–24_ by using Bayesian software. Total number of patients with low AUC_0–24_ = 77 patients (48.7%). Total number of patients with normal AUC_0–24_ = 58 patients (36.7%). Total number of patients with high AUC_0–24_ = 22 patients (13.9%). Normal range of AUC_0–24_ = 400–600 mg·h/L; low AUC_0–24_ < 400 mg·h/L; high AUC_0–24_ > 600 mg·h/L.

Figure 2A indicated that AUC_0–24_ was lower for FN patients (Med = 403) than for non-FN patients (Med = 461), *p* < 0.05. Furthermore, Figure 2B showed a significant decrease in the dose-normalized AUC_0–24_ in FN patients compared to non-FN patients (*p* < 0.05). Furthermore, Table 3 illustrated that 63.9% and 49.2% had vancomycin trough levels that matched the corresponding AUC_0–24_ value in patients with FN and non-FN, respectively. Specifically, patients with FN with AUC_0–24_ of <400 mg·h/L predominantly exhibited agreement with the vancomycin trough method, with the vast majority of patients, 75 patients, having levels less than 10 mg/L. In contrast, AUC_0–24_ greater than 600 mg·h/L showed discordances with the vancomycin trough method, with 10 and 12 patients having trough levels in the lower normal target of 10–15 mg/L and a trough concentration in the higher normal target of 15–20 mg/L, respectively. It is noteworthy that the AUC_0–24_ between 400 and 600 mg·h/L exhibited more discordance with the vancomycin trough method, with 32 of the patients falling within the low vancomycin trough range, i.e., <10 mg/L. However, the remaining showed agreement with normal trough levels, emphasizing the difficulty of achieving optimal dosing in this cohort (Table 3 and Figure 3).

### 2.3. Factors That Contribute to the Estimated AUC_0–24_ in FN Patients

**Figure 4:**
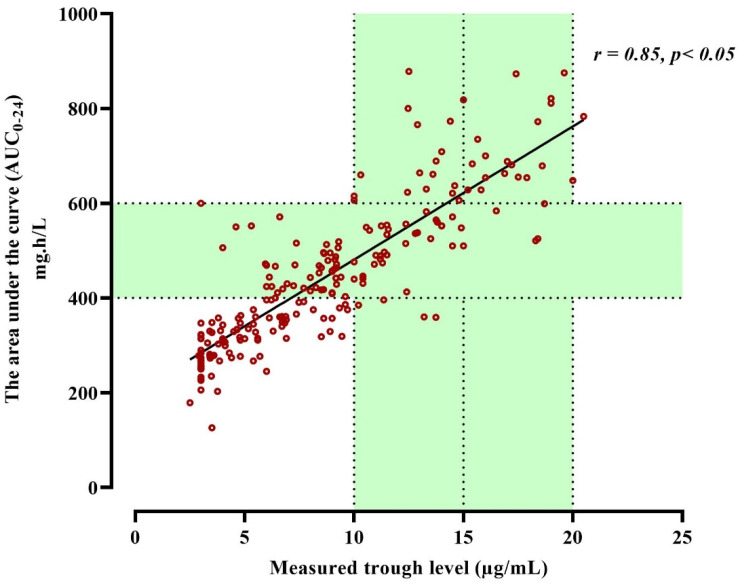
Scatterplot of the vancomycin AUC_0–24_ versus measured trough concentration, r = 0.85.

Figure 4 showed that the correlation between vancomycin AUC and trough concentration was a statistically significant positive correlation (r = 0.85, *p* < 0.05). The overall relationship between AUC_0–24_ and the potential demographics covariates of patients were screened by the multivariate stepwise linear regression analysis to explore a potentially informative covariate. The analysis demonstrated significant correlations for BMI, TDD, CrCl, and trough level with AUC_0–24_ (r = 0.13, 0.26, 0.49, 0.85), respectively *p* < 0.05. The following equation summarizes this relation:

AUC_0–24_ = 15.96 + (23.6 × Trough) + (4.4 × TDD) + (4.4 × BMI) − (0.53 × CrCl)

where AUC_0–24_ is area under the plasma concentration-time curve over the last 24 h dosing interval (mg·h/L), trough is steady-state trough concentration (mg/L), TDD: is the total daily dose (mg/kg/day), BMI: body mass index (kg/m^2^), CrCl: estimated creatinine clearance using the Cockcroft–Gault equation (mL/min).

## 3. Discussion

Several scientific publications have emphasized the benefits of population pharmacokinetic (PK) modeling for enhancing therapeutic drug monitoring (TDM). Bayesian prediction of PK parameters was first utilized for TDM of various drugs, particularly antibiotics with a low therapeutic index, in the late 1970s [8,22]. By utilizing limited concentration measurements, Bayesian predictors and concentration predictions can be employed to estimate the appropriate dose and dosing interval required to achieve a desired exposure endpoint, such as the trough concentration or AUC_0–24_ that is indicative of a favorable benefit-to-risk ratio [23]. The Bayesian approach (Bayesian priori) with trough-only data was associated with 97% (93–102%, *p* = 0.23) accurate AUC_0–24_ estimation as of the gold standard method which includes using the full data concentration [9,24]. In the current study, we estimated AUC_0–24_ based on single-point concentration (trough level). The result showed that AUC_0–24_ was lower for FN patients than non-FN patients 403 vs. 461 mg·h/L. A trough concentration alone may be adequate to predict the AUC_0–24_ using the Bayesian method, but further data from different patient groups are required to prove the validity of employing trough-only measurements. The revised vancomycin TDM guideline states that “a trough concentration alone may be sufficient to estimate the AUC_0–24_ with the Bayesian approach” [4,25]. Moreover, Neely et al. reported a satisfactory performance in estimating AUC_0–24_ using only the trough concentration based on a popPK model created utilizing richly sampled concentration data (approx. 6 sample concentrations during a dosing interval) [26]. Turner et al. reported using the trough concentration to estimate AUC_0–24_ will produce an accuracy (range 0.79–1.03) and bias (range 5.1–21.2%) using the different commercially available Bayesian dose-optimizing software [27]. A recent study by Olney et al. observed that there is a strong correlation between Bayesian two-level and one-level methods (r = 0.93), with an overall 88.5% clinical decision agreement and a low mean difference (MD) between Bayesian and linear AUC methods. They conclude that Bayesian one-concentration approaches may provide an alternate method for predicting AUC_0–24_ and lowering hospital expenditures. Nevertheless, the assumption that one concentration Bayesian equals two concentrations Bayesian is only valid if one assumes that the Bayesian two- concentration technique is the “gold standard” for calculating AUC_0–24_ [28].

FN patients are at risk of receiving suboptimal antibiotics due to pharmacokinetic alterations, which makes frequent proper monitoring necessary to increase efficacy and decrease toxicity. In the current study, comparing the target of the AUC_0–24_ dosing method with the trough-based method showed that only 84 patients (38.7%) reached the target AUC_0–24_ 400–600 mg·h/L, while 77 patients (35.5%) achieved the target trough. In addition, more than 95% of FN patients with low AUC_0–24_ have corresponding trough concentrations <10 mg/L. Additionally, our results demonstrate that 10 patients of FN patients with trough levels in the range of 10–15 and 11 patients with trough concentration in the range of 15–20 were classified as normal trough concentration, while the corresponding AUC_0–24_ was >600 mg·h/L and represented (45.5%) and (50%), respectively. This result can lead to an increased risk of nephrotoxicity, as it is reasonable to assume that misclassification of the trough as therapeutic, without adjustment of the dose or subsequent reduction in the dose, will result in increased vancomycin exposure and a correspondingly higher risk of nephrotoxicity.

On the contrary, from an efficacy point of view, the most significant relationship is reflected in the discordance seen when one method predicts subtherapeutic, and at the same time, the other method estimates it as therapeutic. This study found that the trough method misclassified 32 patients of FN patients as subtherapeutic, while the AUC_0–24_ method considered them in the normal range, representing about 55.2%, Such a variation can prompt the prescriber to increase the dose, and the risk of nephrotoxicity will increase subsequently.

Regarding the PK parameters of vancomycin between HM with/without FN, the finding of the current work illustrates that vancomycin Vd_ss_ and V_p_ in FN-HM patients are significantly lower than in nonneutropenic patients. On the contrary, no differences were detected in Vc and V_dβ_. Furthermore, the results of this study show a significant increase in vancomycin clearance of 23% in patients with FN compared to patients without FN. The present finding for the Vd_ss_ of 0.502 L/Kg seems consistent with other research by Jarkowski et al., which found the Vd_ss_ of 0.59 L/Kg while the V_c_ of 0.23 L/Kg is considered higher than what we reported in this study, V_c_ of 0.14 L/Kg [16]. Haeseker et al. has reported a similar result, reporting that there is no difference in Vd in the FN and non-FN patients, 62 vs. 59 L [20]. Additionally, the current findings support the previous study which proposed that in Japanese patients diagnosed with 22 kinds of cancers, almost one-third as hematological malignancy, Cl_van_ values were affected by renal function, and the adjusted Cl_van_ based on body weight was 0.072 ± 0.028 (L/h/kg) [29]. In line, Izumisawa et al. identified a substantial increase in Cl_van_ in hematologic malignancy patients over non-malignancy patients, 0.07 vs. 0.063 (L/h/kg) [30]. In addition, the current study showed that the t½ of patients with neutropenia was significantly shorter than that for non-FN patients, 5.6 vs. 7.4 h. These results agree with previous studies’ findings, which have demonstrated a shorter t½ of 7.4, 4.9, and 5.6 h in neutropenic patients compared with non-FN 8.9 [19,31,32].

**Figure 5:**
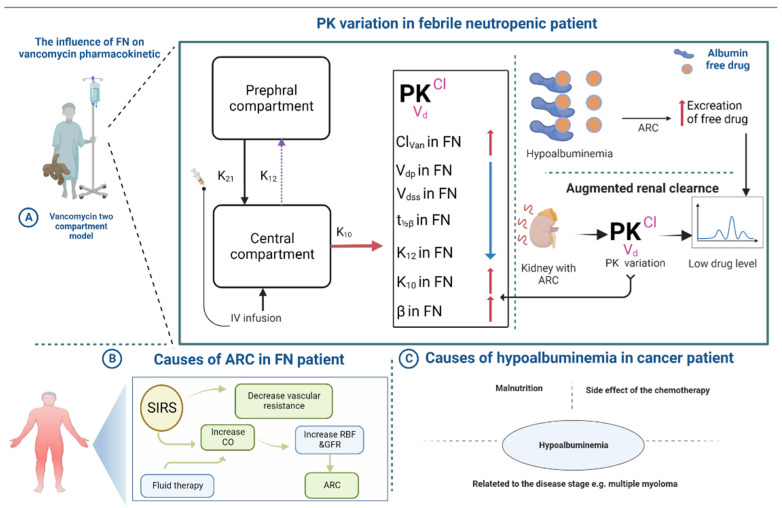
(**A**) Variation in the pharmacokinetics parameters in the FN patient. (**B**) Possible cause of ARC in FN patients. (**C**) The condition that can lead to low albumin level (hypoalbuminemia) in cancer patients. ARC, augmented renal clearance; β, the first-order rate constant for the elimination process; CO, cardiac output; FN, febrile neutropenia; GFR, glomerular filtration rate; k_12_, the first-order transfer rate constant from the central compartment to the peripheral compartment; k_21_, the first-order transfer rate constant from the peripheral compartment to the central compartment; k_10_, the first-order elimination rate constant from the central compartment; Cl_van_, vancomycin clearance; V_dss_, volume of distribution at a steady state; volume of distribution for peripheral compartment V_dp_, volume of distribution during the elimination phase; t_½β_, half-life for the elimination phase.

Among the most important subjects of recent research is the ability to predict the factors substantially responsible for the failure to achieve the desired vancomycin therapeutic goal in patients with FN. Here, we illustrate in Figure 5 the possible hypothetical mechanism behind such failure based on the findings of the current work and our previous work [33]. The first and foremost mechanism assumes that FN exerts its effect by causing variation in the PK parameters of vancomycin, which includes (1) a significant increase in vancomycin clearance, which will lead to a marked short in the half-life of the drug. (2) A significant decrease in the first-order transfer rate constant from the central compartment to the peripheral compartment, k12, might lead to a slight non-significant decrease in the volume distribution from the peripheral compartment. (3) A considerable increase in the first-order elimination rate constant from the central compartment, k10. This would, therefore, decrease the total accumulation and increase the total loss of vancomycin from the body.

A second hypothesis is that FN is significantly associated with ARC. Several studies strongly support that ARC is the primary cause of augmentation of vancomycin clearance [34,35]. The development of systemic inflammatory response syndrome (SIRS) seems to be closely linked to ARC. SIRS is prevalent in critically sick patients with severe infections, trauma, and hematological cancer. Inflammatory mediators generated during SIRS can significantly enhance cardiac output and decrease vascular resistance, resulting in an increase in renal blood flow and GFR. This can be further augmented by the use of high-volume fluid therapy, which is common in patients with cancer, as a part of chemotherapy hydration [17,18,19]. Finally, a third mechanism is hypoalbuminemia, as mentioned early in our previous work. The mechanism of hypoalbuminemia in HMs is unresolved and cannot be definitively attributed to a single, identifiable cause. In addition, literature offers three viable possibilities to explain this. Hypoalbuminemia is particularly common among cancer patients, primarily during therapy or in the last phase. In both instances, malnutrition is a major cause of chronic hypoalbuminemia, but chemotherapy-treated patients may have acute hypoalbuminemia due to acute hepatotoxicity [20]. Hypoalbuminemia may be related to the disease stage. According to Yi-Hsiang Chen et al., the multiple myeloma stage is the primary determinant of albumin level. Almost all patients with stage III myeloma had blood albumin concentrations of 37.0 g/L or less. Thus, hypoalbuminemia is predominantly associated with the degree of myeloma proliferation [21].

In general, the current study has several limitations, including the following: the data collection for this study was performed retrospectively in a single center. Second, the AUC estimation was derived from a single-point concentration, and there was some controversy over the use of the trough to derive valid AUC values. Furthermore, patient outcomes such as cure rate and nephrotoxicity were not evaluated.

## 4. Materials and Methods

The Bayesian-derived AUC and the pharmacokinetic parameters were calculated using a Bayesian software, PrecisePK, which utilizes a single measured vancomycin trough concentration. Several pharmacokinetic models for vancomycin are contained in the PrecisePK^TM^ platform, and the most commonly used model is Rodvold et al., which acts as the default for all noncritically ill patients. This model was developed by Rodvold et al. in 1988 and was designed as a two-compartment model with first-order elimination [36]. The model has been prospectively tested in 45 patients with stable kidney function. The two-compartment model on this platform and population parameters (Bayesian prior values) was used to estimate the Bayesian conditional posterior of patient’s pharmacokinetic parameters informed by the vancomycin trough concentration (s).

### 4.1. Place of Study

This study was carried out in King Abdulaziz Medical City, Princess Norah Oncology Center (PNOC), Jeddah, in the hospital setting from 1 January to 31 December 2020.

### 4.2. Dosing and Monitoring of Vancomycin

Initially, patients started vancomycin at a dose of 15 to 20 mg/kg every 12 to 8 h, depending on their body weight. The dosing interval was chosen and adjusted according to renal function [37]. The clinical pharmacists changed dosing regimens to obtain serum trough concentration at a steady state of 10 to 20 mg/L. The vancomycin trough concentrations were collected 30–60 min before the administration of the fourth dose.

PrecisePK software (Healthware Inc., San Diego, CA, USA) based on the Bayesian theorem was used to calculate AUC_0–24_. The two-compartment model by Rodvold et al. on this platform and the dose data of patients were used to estimate the Bayesian conditional posterior of patient’s pharmacokinetic parameters, estimated AUC_0–24_, and calculated trough.

### 4.3. Study Design and Subjects

All adult HM patients with or without FN who were admitted to the PNOC between 1 January 2017 and 31 December 2020, hospitalized, and received vancomycin doses with a steady-state trough concentration measured before the fourth dose were included. Patients were excluded if they had hemodialysis or required renal replacement therapy or were admitted to the intensive care unit (ICU) or missed patient clinical data. The current work was approved by the ethics committee at King Abdullah International Medical Research Center (KAIMRC) with the approval number NRJ23J/017/01. Vancomycin blood sampling was performed in a steady state for at least 30 min before the fourth dose [26]. FN is defined as “a one-time oral temperature of greater than 38.3 °C (approximately 100.9 °F) or a sustained temperature of greater than 38 °C (100.4 °F) for > 1 h in a patient who has an absolute neutrophil count of less than 500 cells/mL, or an absolute neutrophil count expected to decrease to less than 500 cells/mL within 48 h” [38]. The baseline data of patients, which included age, sex, weight, and serum creatinine, were obtained before starting vancomycin’s first dose. The Cockcroft–Gault equation was used to calculate creatinine clearance [39]. The Bayesian approach was used to estimate the vancomycin AUC_0–24_. The dates, timings of vancomycin dose administration, vancomycin trough concentration, age, gender, serum creatinine weight were entered into the PrecisePK software to calculate AUC_0–24_. Once data entry was completed, the AUC_0–24_ and individual pharmacokinetics were obtained from the software for each patient.

### 4.4. Endpoints

- To evaluate the difference in estimated AUC_0–24_ between FN and non-FN HM patients and determine the percentage of patients who achieved optimal AUC_0–24_ by using Bayesian software (posterior).
- To assess the difference in vancomycin PK parameters between FN and non-FN HM patients.
- To identify factors that contribute to the estimated AUC_0–24_ in FN patients.

### 4.5. Bioassay

Serum vancomycin concentrations were analyzed using an immune-chemiluminescence immunoassay (Archetict i2000, Abbott, Park, IL, USA) in each patient as part of the standard normal protocol for the treatment of patients (Abbott, Abbott Park, IL, USA). In most cases, blood tests were obtained between 30 and 60 min before the fourth dose, with the aim of maintaining that a steady state was reached. The minimum detectable concentration was established at 3.0 mg/L, while the maximum detectable concentration was established at 100.0 mg/L.

### 4.6. Statistical Analysis

The statistical package for the social sciences (SPSS) version 26.0 (SPSS Inc., Chicago, IL, USA) was used for statistical analysis in this study. All continuous data were tested for normality using a histogram and the Shapiro–Wilk test. Demographic data were expressed as frequencies and percentages for categorical variables, where continuous variables were presented as the mean ± SD or the median (interquartile range) when applicable. The Student *t*-test was used to compare normally distributed variables between two groups, while the Mann–Whitney for those not normally distributed. The association between the two groups was performed using a contingency table analysis with a χ^2^ test. The multivariate stepwise linear regression analysis was used to determine the overall relationship between AUC_0–24_ and the potential covariates. Categorical matching was used to determine the clinical decision agreement and characterize the concordance for which the trough method and AUC_0–24_ estimations resulted in classification as subtherapeutic, therapeutic, or supratherapeutic. The clinical decision agreement gives an estimate of the degree to which the different methods would have affected clinical decision-making in practice (dose modification, i.e., increase, decrease, or no change). A *p*-value of < 0.05 was considered statistically significant.

## 5. Conclusions

The findings of this work conclude that AUC_0–24_ was lower for FN patients than non-FN patients 403 vs. 461 mg·h/L. Furthermore, the neutropenic patient has a higher Cl_van_, a shorter t½, and a lower Vd_ss_, which can explain how FN could affect the vancomycin trough concentration. Based on these findings, it may be necessary to consider a higher or more frequent dose of vancomycin for patients with FN to achieve the optimal vancomycin AUC_0–24_. It is recommended that further prospective research be conducted to assess neutropenia effects with more than one sample. This will easily facilitate the interpretation of the kinetic-guided dose calculation for such patients.

## Figures and Tables

**Table 1:** Baseline demographics and clinical characteristics.

Variable	FN (n = 158)	Non-FN (n = 59)	*p*-Value
Gender			
Male, n (%)	87 (55.10)	38 (64.40)	0.2
Female, n (%)	71 (44.90)	21 (35.60)	0.2
Age, years, (mean ± SD)	44.96 ± 15.64	43.34 ± 16.55	0.51
BMI, (mean ± SD)	27.11 ± 6.47	28.57 ± 9.29	0.25
CrCl, mL/min, (mean ± SD)	125.99 ± 42.91	102.16 ± 36.59	<0.05
TDD, mg/kg/day (mean ± SD)	31.11 ± 41.48	30.48 ± 71.49	0.59
Albumin concentration g/L (mean ± SD)	31.79 ± 4.8	31.47 ± 5.5	0.67
ARC n (%)	75 (47.4)	15 (25.4)	
Indication of vancomycin			
Documented infection, n (%)	7 (4.40)	26 (44.10)	
Empirical therapy, n (%)	151 (95.60)	33 (55.90)	
Malignancy Type, n (%)			
Leukemia, n (%)	140 (64.50)		
Lymphoma, n (%)	62 (28.60)		
Multiple myeloma, n (%)	15 (6.90)		
Trough concentration mg/L (mean ± SD)	8.2 3 ± 4.58	11.12 ± 5.71	<0.05
<10 mg/L	107 (67.7%)	33 (33.9%)	
≥10–<15 mg/L	38 (24.1%)	14 (23.7%)	
≥15–<20 mg/L	13 (8.2%)	12 (20.3%)	
AUC_0–24_ (IQR, 25th–75th percentile)	403 (314–525)	461 (363.5–614.5)	<0.05
Low AUC_0–24_ < 400 mg·h/L n (%)	78 (48.7)	19(32.2)	
Normal AUC_0–24_ 400–600 mg·h/L n (%)	58 (36.7)	26 (44.1)	
High AUC_0–24_ > 600 mg·h/L n (%)	22 (13.9)	14 (23.7)	
Dose-normalized AUC_0–24_ mg·h/L/mg/kg (mean ± SD)	14.2 ± 5.2	17.2 ± 6.4	<0.05

ARC, augmented renal clearance; CrCl, creatinine clearance; FN, febrile neutropenia; TDD, total daily dose.

**Table 2:** The difference in individual pharmacokinetics parameters between FN and non-FN patients.

	Patients with FN	Patients without FN
PK Parameters	Mean ± SD/Median	Mean ± SD/Median
Vd_β_ (L/kg)	0.629 ± 0.082 ^b^	0.629 ± 0.088
CL_van_ (L/h/kg)	0.078 ± 0.030 ^a^	0.061 ± 0.026
V_p_ (L/kg)	0.352 ± 0.068 ^a^	0.382 ± 0.072
Vd_ss_ (L/kg)	0.502± 0.078 ^a^	0.531 ± 0.083
k_10_ (h^−1^)	0.504 ± 0157 ^a^	0.389 ± 0.143
k_12_ (h^−1^)	1.071 ± 0.161 ^a^	1.150 ± 0.159
β (h^−1^)	0.120 (0.09–0.15) ^a^	0.100 (0.07–0.12)
t_½, β_ (h)	5.630 (4.41–7.17) ^a^	7.400 (5.38–9.60)
V_c_ (L/kg)	0.144 (0.14–0.17) ^b^	0.141 (0.14–0.17)
α (h^−1^)	1.900 (1.86–1.95) ^b^	1.900 (1.87–1.98)
t_½,α_ (h)	0.370 (0.35–0.37) ^b^	0.370 (0.35–0.37)

Values are expressed as the means ± SD or the median [IQR, 25th–75th percentile]; n = 217; ^a^ *p* < 0.05 compared with the corresponding non-FN group values; ^b^ *p* > 0.05 compared with the corresponding non-FN group values; by the Student *t*-test or the Mann–Whitney U test.

**Table 3:** The clinical decision agreement between FN and non-FN patients.

***Febrile neutropenic patients***					
AUC_0–24_	*Vancomycin trough level (mg/L)*				
		*<10*	*≥10–<15*	*≥15–≤20*	*Total*
	*<400*	75 (96.1%)	3 (3.9%)	0	78
	*400–600*	32 (55.2%)	25 (43.1%)	1 (1.7%)	58
	*>600*	0	10 (45.5%)	12 (54.5%)	22
	*Total*	107	38	13	158
	***Total matched = 101/158 = 63.9%***				
***Non-febrile neutropenic patients***					
	*Vancomycin trough level (mg/L)*				
AUC_0–24_		*<10*	*≥10–<15*	*≥15–≤20*	*Total*
	*<400*	*18* (94.7%)	*1* (5.3%)	*0*	*19*
	*400–600*	*15* (57.7%)	*7* (26.9%)	*4* (15.4%)	*26*
	*>600*	*0*	*6* (42.80%)	*8* (57.2%)	*14*
	*Total*	*33*	*14*	*12*	*59*
	***Total matched = 29/59 = 49.2%***				

## Data Availability

The datasets used and/or analyzed during the present study are available from the corresponding author on reasonable request.
